# Study on the Progress of Neural Mechanism of Positive Emotions

**DOI:** 10.1515/tnsci-2019-0016

**Published:** 2019-04-23

**Authors:** Jie Yin

**Affiliations:** 1 School of Educational Science, Hengyang Normal University, Hengyang 421002, China

**Keywords:** cognitive process, positive emotions, neural mechanism

## Abstract

Positive emotions refer to emotions accompanied by pleasant feelings, such as happiness, interest, satisfaction, pride, and love, which are generated by individuals in response to internal and external stimuli and events. Positive emotions are conscious processes that involve a variety of components, such as a pleasant experience, facial/body expressions, evaluations, and especially behavioural plans and activation states. People's cognitive process is often accompanied by emotions, and the influence of positive emotions on cognitive activities has gradually become the focus of research in recent years. This study constructs a regulation model and an assessment model of positive emotions, and analyses the neural mechanisms of the effects of dopamine substances on human positive emotions.

## 1 Introduction

Previous rich research results have shown that positive emotions play a certain role in promoting various cognitive processes, such as attention, memory, mental rotation, problem solving and social cognition. According to a large number of previous studies, the cognitive promotion effect of positive emotions can be summarized as broadening the cognitive scope and promoting cognitive flexibility.

Positive emotion refers to the emotion accompanied by pleasure feelings generated by an individual due to internal and external stimuli and events to meet individual needs, including happiness, interest, satisfaction, pride, love, etc. Positive emotion is a conscious process that includes pleasure experience, facial/body expression, evaluation, especially behavioral planning and activation state. People’s cognitive process is often accompanied by emotions, and the influence of positive emotions on cognitive activities has gradually become a research hotspot in recent years.

### 1.1 Cognitive Science

In the early stage of human cognition, people put their consciousness to the outside world and thought about what the origin of the world was. After modern times, the object of philosophical study turns to the subject itself and studies how to understand the possible problem, which is modern epistemological philosophy. After the middle of the 20th century, philosophy began to turn its eyes to the language of the intermediate link between the subject and the object, which is the contemporary western philosophy of language. In the study of linguistics and philosophy of language, linguists and philosophers of language have found that language is a reflection of the mind, and the mind is a function of the brain. Many of them have moved from the study of language to the study of mind and cognition.” Language is the mirror of the soul,” Chomsky said. “Language is a fundamental function of the human mind,” said sale. After the mid-1970s, the object of philosophy naturally turned to the human mind itself. According to sale, “the most important development in cognitive science is the shift of cognitive scientists from computational models of cognitive science to models of cognitive neuroscience. This shows that the brain as the basis of cognition has replaced the digital computer as the basis of cognition. We see the neurobiological brain as the basis of human cognition, and this is a very important shift. He added: “the most promising area of research is cognitive neuroscience, not just brain micro nanotechnology.

**Figure 1:**
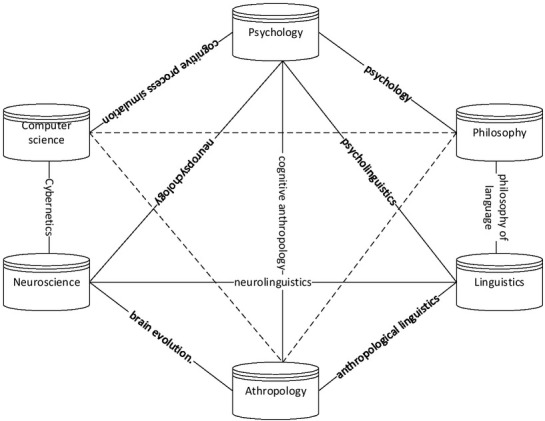
Structure of the cognitive science discipline

Cognition is the process and activity in which the brain and nervous system produce the minds. Generally speaking, animals with brain and nervous system have a certain degree of mind. Cognitive science is a science that takes cognitive process and rules as research object, and cognition involves learning, memory, thinking, understanding, and other behaviours that occur in the cognitive process ^[1]^. The structure of the previously internationally recognized discipline of cognitive science is shown in Figure 1.

During the development of cognitive science, 6 new development directions are produced within the original 6 supporting disciplines, including mental philosophy, cognitive psychology, cognitive linguistics (or language and cognition), cognitive anthropology (or referred to as culture, evolution and cognition), artificial intelligence, and cognitive neuroscience. These 6 emerging disciplines are the six major branches of cognitive science. These 6 supporting disciplines intersect with each other to produce 11emerging interdisciplinary subjects: (1) cybernetics; (2) neurolinguistics; (3) neuropsychology; (4) cognitive process simulation; (5) computational linguistics; (6) psycholinguistics; (7) psychological philosophy; (8) language philosophy; (9) anthropological linguistics; (10) cognitive anthropology; and (11) brain evolution.

The important progress of cognitive science has benefited from the development of brain science, which in turn has benefited from the great progress of brain imaging technology. Computer tomography (CT), magnetic resonance imaging (MRl), functional magnetic resonance imaging (fMRl) and positron emission tomography (PET) are widely used in the research of brain and neuroscience, which promotes the development of brain and neuroscience ^[2]^. These developments in brain science have prepared conditions for us to reveal the secrets of the mind.

### 1.2 Positive emotions

**Figure 2:**
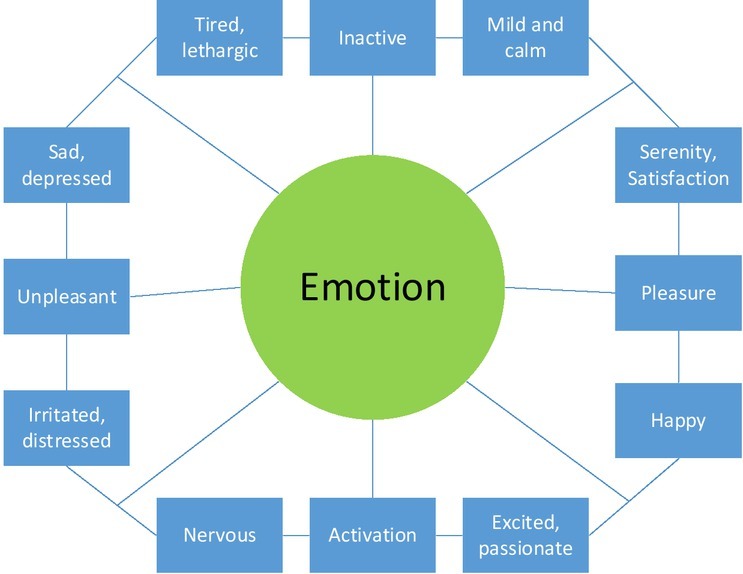
Two-dimensional model of emotions

The “positive” in positive emotions is derived in contrast with “negative” in negative emotions. According to Russell’s circumplex model (i.e., the two-dimensional theory of emotions), emotions are considered to contain two dimensions, valence and arousal. The valence dimension is divided into pleasantness-unpleasantness, and the arousal degree is divided into high-low (as shown in Figure 2) ^[3]^. According to this model, people preliminarily define the positive emotion and the negative emotion.

## 2 Positive emotional function

### 2.1 Positive emotions expand cognitive range

Positive emotions have a wide range of effects on cognitive processes. Many previous studies have found that people who experience positive emotions exhibit unusual, flexible, creative, integrated, information-open-minded, and effective thinking patterns. The promotion effect of positive emotion on cognition is not only reflected in the speed and accuracy of processing, but also can change the cognitive function in categories and levels, and cause the change of cognitive structure in information processing, that is, it affects the cognitive process qualitatively and quantitatively.

Positive emotions such as happiness, interest, satisfaction and so on can extend the individual’s instantaneous thinking activity sequence. Negative emotions generally narrow the sequence of individual instantaneous thinking activities and the scope of individual’s cognition, so that the individual in the situation at that time only produce some specific behaviour and the individual body energy is mobilized to deal with specific environmental challenges. Positive emotions can induce individuals to break through certain restrictions and generate more ideas under normal conditions. They can expand the individual’s attention range, enhance cognitive flexibility, and update and expand cognitive maps of the individuals ^[4]^.

### 2.2 Personal resources built by positive emotions

Positive emotions cannot only improve the efficiency of solving cognitive problems, but also facilitate the solution of interpersonal problems and negotiations. The emotions cannot only create a friendly atmosphere for negotiations, but also enable negotiators to come up with more problem-solving strategies due to the increased cognitive flexibility under positive emotions. They can bring about friendly behaviour such as help, facilitate interpersonal relationship and expand interpersonal resources. They can improve people’s coping abilities and promote social adaptation.

For example, in an organizational environment, positive emotions can affect managers’ decision-making, conflict resolution, group behaviour, perception of work tasks, etc. These studies show that positive emotions increase a person’s ability to organize thoughts in a variety of ways. In these studies, subjects were randomly assigned to neutral emotions and positive emotions groups, and various simple methods were used to induce positive emotions and complete different tasks to test creative problem solving. The results were relatively consistent.

### 2.3 The expansion of positive emotions

**Figure 3:**
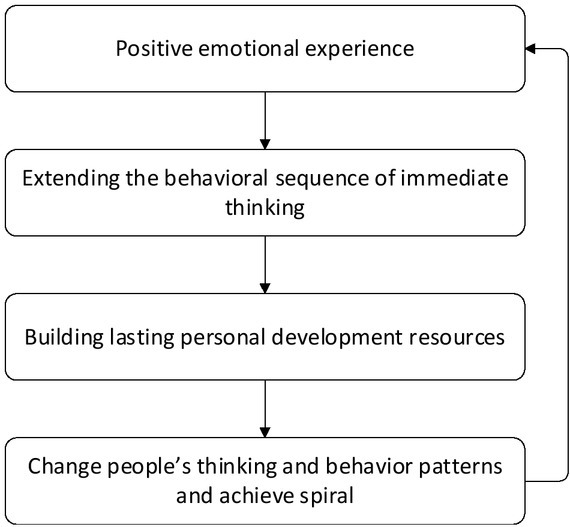
The extended effect of positive emotions

Positive emotions such as happiness, interest, satisfaction and so on can extent the individual’s instantaneous thinking activity sequence, and can build individual resources, including physical resources such as physical skills, health, intellectual resources knowledge, psychological theory, executive control, interpersonal resources and friendship, social support networks, and psychological resources, such as psychological resilience, optimism, creativity ^[5]^. Figure 3 graphically illustrates the process of “the expansion and utility of positive emotions,” which is a spiraling process.

## 3 The basic model of positive emotions

### 3.1 The model of the adjustment process of positive emotions

Past models of the relationship between positive emotions and cognition suggest that positive emotions lead to cognitive broadening and that positive emotions lead to increased cognitive flexibility. However, they sometimes diverge in their predictions, with a widening view predicting the expansion of attention (overall preference) that the cognitive flexibility view does not. Gable and harmon-jones recently proposed a motivational dimension model, suggesting that the reason for the narrowing/broadening of attention and cognition by emotion is motivational intensity rather than emotional valence. Only positive emotions that approach low motivational intensity can broaden the cognitive process and flexibility, while positive emotions that approach high motivational intensity narrow the cognitive process and flexibility. Past studies have concluded that positive emotions promote cognition because the motivation intensity of induced positive emotions is relatively low, which can broaden the cognitive process. The method of the trigger positive emotions to participants unexpected gifts (such as candy, etc.), look at the pictures lead to positive emotions or comedy movies, imagine happy event, induced by positive emotional words and music, report success in the task, and so on, is usually caused by happy, satisfaction, pleasure, etc., all these emotions are “goals” or “irrelevant” low motivation intensity of positive emotions.

**Figure 4:**
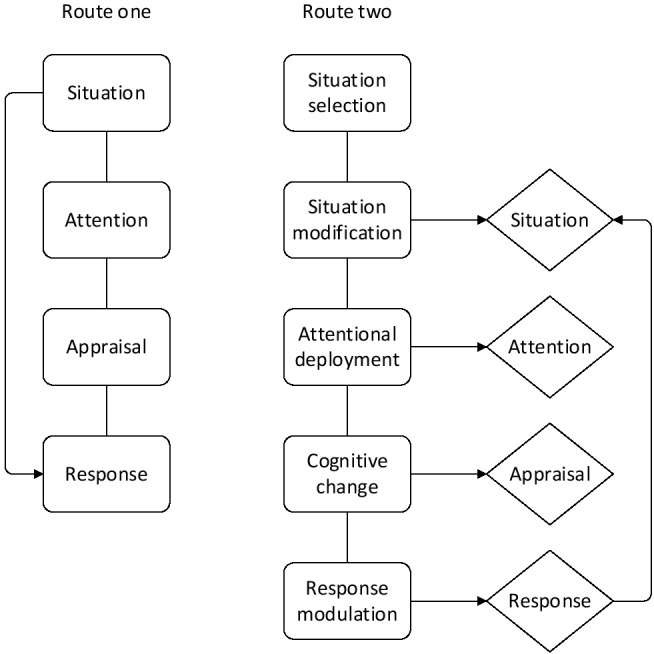
Modulation process model

Based on the process model of emotion generation in the previous section, emotion regulation can act on any stage of emotion generation. Therefore, the emotional adjustment process can be divided into five categories: situation selection, situation modification, attentional deployment, cognitive change and response modulation ^[7]^, which act on different emotion generation stages, as shown in Figure 4 ^[6]^.

### 3.2 Evaluation model of positive emotions

The process model of emotion regulation mainly focuses on the execution process of emotion regulation, that is, the process in which the individual adopts a specific strategy to regulate emotion. However, some recent studies have suggested that emotion regulation actually involves multiple stages of psychological processes. For example, after contain emotion is generated; the individual shall determine whether the emotion needs to be regulated, choose the appropriate emotion regulation strategy, and then adopt the specific strategy for regulation. The basic idea of an extended model of emotion regulation is that, like other types of emotions, emotions involve the process of assessment. Specifically, there are four components in the assessment system: world, perception, assessment, and action ^[8]^.

**Figure 5:**
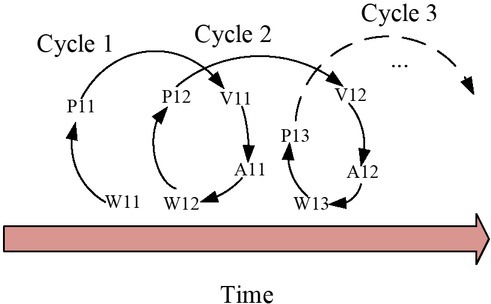
Evaluation cycle process

Through the comparison of the assessment system with the previous process model of emotion regulation, it is not difficult to find that there is a one-to-one relationship between them: situation-world, attention-perception, appraisal- assessment and response - action. The most important part of the assessment system is its dynamics. First, the behaviour produced by each appraisal cycle is targeted at one or more aspects of the “world” in the appraisal cycle. When the “world” part of the cycle changes, the perception of the “world” cycle by the subsequent appraisal cycle will change, resulting in a change in the subsequent appraisal and action (see Figure 5) ^[9]^. The cycle of the appraisal system stops only when the difference between the individual’s target state and the changed world is below a certain threshold.

## 4 The neural mechanism of positive emotional cognition promoting effect

### 4.1 Dopamine properties

**Figure 6:**
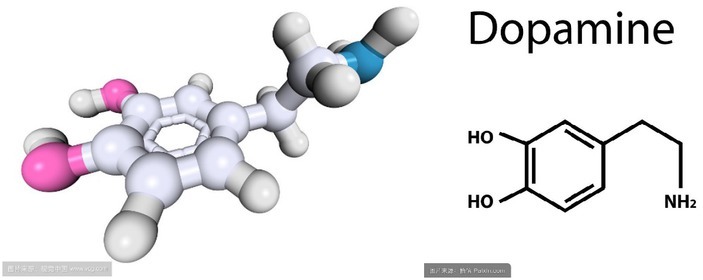
Molecular structure of polyamine bar

Dopamine is a neurotransmitter that helps cells to transmit pulsed chemicals. This brain secretion is associated with human eroticism and feelings, which conveys messages of excitement and happiness. It is a precursor of NA, and a key neurotransmitter in the hypothalamus and pituitary gland. The concentration of dopamine in the nervous system is influenced by mental factors, there is axon connection and interaction between GnRH of the nerve endings and dopamine, and dopamine can inhibit the secretion of GnRH. The neuronal substance dopamine in the midbrain directly affects people’s emotions. In theory, the addition of this substance can make people excited, but it can be addictive, too. Dopamine appears in the forebrain and basal ganglia, of which the latter responsible for dealing with fear. However, because of dopamine, the feeling of fear is replaced, thus there are many people who are addicted to dopamine ^[10]^. Similarly, the role of positive emotions in the human body is also directly related to dopamine, whose structural model is shown in Figure 6.

Ashby et al. put forward the neuropsychological theory that positive emotion promotes cognition, believing that many effects of positive emotion on behaviour and cognition (such as promoting creative problem solving, improving cognitive flexibility, etc.) are mediated by the dopamine system. The more important mesolimbic system is made up of dopamine-producing cells projected into the VTA, which is associated with reward and motivation. Many of the important dopamine projections may be the neural mechanisms responsible for the cognitive effects of positive emotions. For example, studies have shown that improvements in creative problem solving are due to positive emotional conditions associated with increased dopamine levels in the frontal cortex. The theory has two hypotheses about VTA projection: first, positive emotions may alter the processing of any structure that receives a direct VTA projection. Second, behavioural changes resulting from positive emotions are unlikely to be mediated by structures that do not receive VTA direct projections.

Experiments have shown that decreased dopamine levels are associated with anhedonia (emotional apathy or anhedonia) rather than negative emotions. Stressful or anxiety-inducing events that cause people to experience negative emotional states actually seem to increase dopamine levels in specific brain regions. So do high and low dopamine levels explain the motivational dimensional model to some extent? Ashby speculated that the correlation between dopamine levels and task performance might be inverted u-shaped, so any increase or decrease in the optimal level of dopamine would impair performance. Additional research has shown that very high levels of dopamine are associated with three characteristics of borderline personality disorder - mood disorders, impulsive behaviour, and cognitive-perceptual deficits. It can be seen from the above evidence that dopamine may have an optimal level when mediating the effect of positive emotion on cognition, and according to the motivational dimension model, the promotion effect of positive emotion on cognition may also present an inverted u-shaped distribution in the intensity of motivation approach. So whether there is a relationship between dopamine levels and the motivational approach intensity of positive emotions is not studied in the available literature.

In fact, when comparing the moderately positive emotion and neutral emotion induced, not only the increase of dopamine level was found in various brain regions, but also the peripheral blood circulation system, such as the dopamine concentration in serum, showed a significant increase. In addition, they found increased activity in NK cells, a type of immune cell associated with positive emotions. Be aware of the effects of neurotransmitters and neuromodulators on positive emotions in addition to dopamine.

Many of the effects of positive emotions on behaviour and cognition (such as promoting creative problem solving, improving cognitive flexibility, etc.) are mediated by the dopamine system. The more important mesolimbic system consists of dopamine-producing cells projecting into the marginal and ventral tegmental area of the cortex, which are associated with reward and motivation.

### 4.2 The neural mechanism of positive emotions

**Figure 7:**
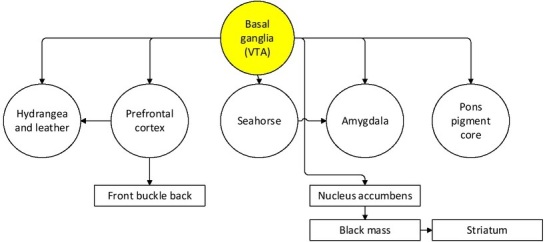
Dopamine projections in human brain

Many important projections of dopamine (see Figure 7) are the neural mechanisms by which positive emotions affect cognition. For example, studies have shown that improvements in creative problem solving are due to positive emotional conditions associated with increased dopamine levels in the frontal cortex. The theory has two assumptions about VTA projection: First, positive emotions may alter the processing of any structure that receives direct projections from the VTA. Second, behavioural changes caused by positive emotions are unlikely to be mediated by structures that do not receive direct projection from the VTA ^[11]^.

Experiments have shown that a drop in dopamine levels is associated with lack of pleasure (apathy or lack of pleasure) rather than with negative emotions. Stressful events that cause people to develop negative emotional states actually seem to increase the level of dopamine in certain regions of the brain. The correlation between the levels of dopamine and task performance is inverted U-shaped, so an increase or decrease in dopamine from the optimal level can impair performance. In addition, studies have shown that high levels of dopamine are associated with three characteristics of borderline personality disorder, such as emotional disorders, impulsive behaviour, and cognitive-perceptual defects, that’s, high dopamine levels have a detrimental effect on cognition. It can be seen from the above evidence that dopamine may have an optimal level in mediating the effect of positive emotions on cognition. However, according to the motivation dimension model, the positive effect of positive emotions on cognition may also show an inverted U-shaped distribution in the approach- motivation intensity.

In fact, the comparison of induced moderate positive emotions and neutral emotions shows that an increase in dopamine levels is not only found in the brain regions, but also in the peripheral blood circulation system. For instance, the concentration of dopamine in the serum shows a significant increase. In addition, there is an increase in the activity of immune cells - NK cells (natural killer cells) under positive emotions. The effects of other neurotransmitters and neuromodulators on positive emotions shall be noted in addition to dopamine.

## 5 Conclusions

At present, there are still some problems in the research on the effect of positive emotions on cognitive enhancement. In the process of inducing positive emotions, the positive emotions induced by different methods are not only not quantified or even qualitatively unclear. As Gable et al. pointed out, most of the inducing methods in past studies produced positive emotions with low motivation, which is because the motivation dimension of positive emotions has not been taken into account in the past.

Based on cognitive science, this study briefly describes the basic characteristics and functions of positive emotions, constructs the regulation process model and assessment model of positive emotions, and probes into the role of dopamine in the process of emotion generation, intervention and development. The study on the effects of positive emotions on cognition and its neural mechanisms can help to understand normal and abnormal neuropsychological functions. For example, a decrease in positive emotions may make it difficult for people to perform tasks that rely on executive function, which is a common phenomenon in depressed individuals. This study has a great significance on the education cause. Teachers shall treat each student as an individual with creative potential, and provide an environment that helps them to exert their creativity, one of which is the emotional state of pleasure.
